# Anti-atherosclerosis mechanisms associated with regulation of non-coding RNAs by active monomers of traditional Chinese medicine

**DOI:** 10.3389/fphar.2023.1283494

**Published:** 2023-11-06

**Authors:** Guoqing Liu, Liqiang Tan, Xiaona Zhao, Minghui Wang, Zejin Zhang, Jing Zhang, Honggang Gao, Meifang Liu, Wei Qin

**Affiliations:** ^1^ School of Pharmacy, Shandong University of Traditional Chinese Medicine, Jinan, Shandong, China; ^2^ School of Pharmacy, Jining Medical University, Rizhao, Shandong, China; ^3^ Department of Nasopharyngeal Carcinoma, Sun Yat-sen University Cancer Center, Guangzhou, Guangdong, China; ^4^ School of Pharmacy, Weifang Medical University, Weifang, Shandong, China; ^5^ School of Pharmacy, Binzhou Medical University, Yantai, Shandong, China

**Keywords:** non-coding RNAs, traditional Chinese medicine, atherosclerosis, cardiovascular diseases, active monomer

## Abstract

Atherosclerosis is the leading cause of numerous cardiovascular diseases with a high mortality rate. Non-coding RNAs (ncRNAs), RNA molecules that do not encode proteins in human genome transcripts, are known to play crucial roles in various physiological and pathological processes. Recently, researches on the regulation of atherosclerosis by ncRNAs, mainly including microRNAs, long non-coding RNAs, and circular RNAs, have gradually become a hot topic. Traditional Chinese medicine has been proved to be effective in treating cardiovascular diseases in China for a long time, and its active monomers have been found to target a variety of atherosclerosis-related ncRNAs. These active monomers of traditional Chinese medicine hold great potential as drugs for the treatment of atherosclerosis. Here, we summarized current advancement of the molecular pathways by which ncRNAs regulate atherosclerosis and mainly highlighted the mechanisms of traditional Chinese medicine monomers in regulating atherosclerosis through targeting ncRNAs.

## Graphical Abstract

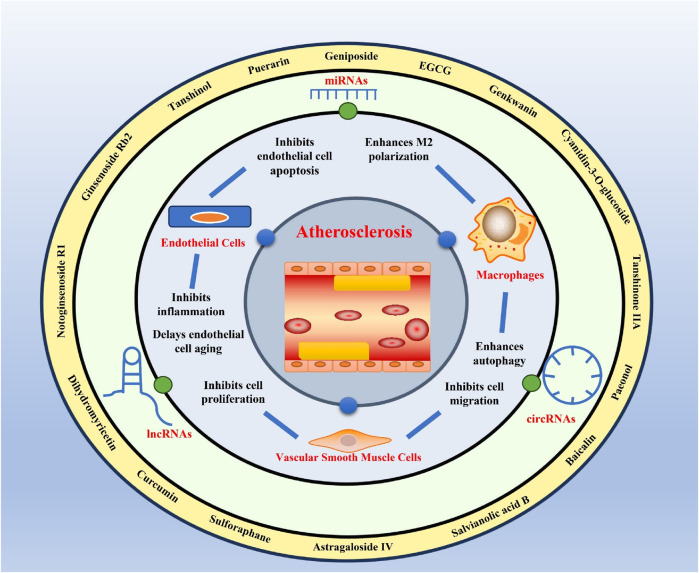

## 1 Introduction

Atherosclerosis is characterized by fibro-fat lesions on the walls of arteries with extremely high morbidity and mortality rate (Libby et al., 2019). Atherosclerosis is considered to be an important pathological basis for cerebrovascular and cardiovascular diseases such as cerebral infarction, coronary heart disease and myocardial infarction (Libby, 2021). There are many causes of atherosclerosis, such as inflammatory reactions (Zhu et al., 2018), cell death and aging (Bazioti et al., 2022), and endothelial to mesenchymal transition (Liang et al., 2022), among which chronic inflammation is the reason that has been frequently studied in the past few years. At the molecular level, telomere damage, genomic DNA damage, and mitochondrial DNA damage accumulate in vascular endothelial cells, which induce endothelial cell aging and chronic inflammation. Persistent inflammation results in increased accumulation of lymphocytes and macrophages, leading to atherosclerosis (Wang and Bennett, 2012; Okuyama et al., 2015; Ruparelia and Choudhury, 2020). The pathogenesis and therapeutic targets of atherosclerosis have long been the focus in the field of cardiovascular researches. Statins, inhibitors of the hydroxymethylglutaryl-CoA (HMG-CoA) reductase enzyme, are powerful cholesterol lowering medications and the most commonly used clinical drugs for the prevention and treatment of atherosclerosis (Okuyama et al., 2015). Statins can reduce morbidity and mortality in patients with cardiovascular diseases. However, statins may affect the drug-drug interactions because different safety and tolerability, especially when in combination with other cardiovascular drugs, which will lead to increased risk of statin-associated hepatotoxicity and myopathy (Bellosta and Corsini, 2018). Therefore, it is urgent to discover new therapeutic targets and new drugs for atherosclerosis.

RNAs in mammalian cells are complex, some of which have the function of encoding proteins, but some of which lack this function. At present, the RNAs that lack the function of encoding proteins are named as non-coding RNAs (ncRNAs), of which the most studied are microRNAs (miRNAs), long non-coding RNAs (lncRNAs), and circular RNAs (circRNAs) (Lu and Rothenberg, 2018; Chen et al., 2021a; Bridges et al., 2021). NcRNAs have been proved to play important regulatory roles in pathogenesis of atherosclerosis through affecting inflammatory reaction, cell activation and proliferation, and lipid metabolism (Feinberg and Moore, 2016; Aryal and Suarez, 2019). Nowadays, therapeutic strategies targeting ncRNAs have entered the clinical testing phase for the treatment of cancers and have been considered as an attractive approach for the treatment of atherosclerosis.

Since ancient times, numerous herbal medicines have been used for the treatment of atherosclerosis-related diseases and decoction is the main form of traditional Chinese medicine used in clinic. With the development of separation technology, it has become possible to separate more pharmacologically active monomers from traditional Chinese medicine. This allows researchers to conduct pharmacological studies on the specific monomers rather than the whole of medicinal plants. At present, studies have found that many active monomers of traditional Chinese medicine have positive effects on atherosclerosis, such as saponins (Luo et al., 2022), flavonoids (Park et al., 2006) and alkaloids (Li et al., 2021d). For example, berberine, an active ingredient extracted from *Berberis aristata* DC., can activate PPAR-γ pathway in macrophages, resulting in decreased expressions of inflammatory factors like monocyte chemoattractant protein-1 (MCP-1) and tumor necrosis factor-α (TNF-α) (Chen et al., 2008). Another study found that hydroxysafflor yellow A, a natural compound from *Carthamus tinctorius L.*, exerts protective effects on atherosclerosis by suppressing vascular endothelial cell dysfunction, vascular smooth muscle cell proliferation and migration, foam cell formation, and platelet activation (Xue et al., 2021). These active monomers of traditional Chinese medicine are predicted to have high therapeutic potential in atherosclerosis treatment. However, the specific drug targets of these active ingredient are not fully understood, which limits clinical application.

Recently, more and more studies have found that ncRNAs are the key mediators of the pharmacological effect of traditional Chinese medicine. Here, we summarized current advances of mechanisms of ncRNAs in regulating atherosclerosis. Furthermore, we highlighted the current advances in the active monomers of traditional Chinese medicine which have atheroprotective effects by regulating ncRNAs.

## 2 The role of ncRNAs in regulating atherosclerosis

### 2.1 miRNAs and atherosclerosis

MiRNAs (typically 20–25 nucleotides) are single-stranded RNA molecules that can bind to complementary sequences within the 3′ untranslated region of mRNA targets. Once the miRNA binds to the mRNA, it can degrade the mRNA via cleavage or inhibit the translation of mRNAs into proteins (Winter et al., 2009; Thum and Mayr, 2012). MiRNAs are the most studied ncRNAs in atherosclerosis and have been shown to regulate the fate and function of atherosclerosis associated cells, including endothelial cells, inflammatory cells, and vascular smooth muscle cells (VSMCs). MiRNAs can affect endothelial cell function by exacerbating senescence of endothelial cells, which is considered as a key mechanism of atherosclerosis (Menghini et al., 2009; Fiedler and Thum, 2016). There are many miRNAs involved in the regulation of endothelial cell senescence, such as miR-146a and miR-217 (Wang et al., 2021; Xiao et al., 2021). Studies have found that mesenchymal stem cell-derived extracellular vesicles attenuate endothelial cell senescence by regulating miR-146a/Src signaling (Xiao et al., 2021). MiR-217 can also promote endothelial cell senescence through the SIRT1/p53 signaling pathway (Wang et al., 2021). In addition, miRNAs can control the inflammatory state of the vasculature by affecting leukocyte activation and infiltration (Perez-Sanchez et al., 2017; Pankratz et al., 2018). In the setting of atherosclerosis, miR-126 promotes macrophage polarization to the M2 phenotype by downregulating VEGFA and krüppel-like factor 4 (KLF4) (Shou et al., 2023). MiRNAs have also been shown to affect foam cell formation and subsequent plaque formation (Eken et al., 2017; Maitrias et al., 2017). MiR-302a has been shown to promote the formation of foam cells and increase the outflow of cholesterol in macrophage by increasing ATP-binding cassette transporter A1 (ABCA1) activity (Meiler et al., 2015). In addition, the function of VSMCs can also be regulated by miRNAs. For example, miR-146b-5p reduces the expression of its target genes Bag1 and Mmp16, thereby affecting the proliferation and migration of VSMCs during atherosclerosis (Sun et al., 2020). A study also found that miR-374 may be a potential biomarker for the diagnosis of atherosclerosis, and overexpression of miR-374 promotes the proliferation and migration of VSMCs (Wang et al., 2020b). MiR-663 can target high mobility group AT-hook 2 (HMGA2) to inhibit the proliferation of VSMCs, thereby delaying the development of atherosclerosis (Deng and Li, 2022). In conclusion, miRNAs regulate atherosclerosis through affecting the function of endothelial cells, macrophages, and VSMCs.

### 2.2 lncRNAs and atherosclerosis

LncRNAs are ncRNAs longer than 200 nucleotides (Di Mauro et al., 2018), which are abnormally expressed in many pathological tissues (Li et al., 2020; Zang et al., 2020). Unlike miRNAs, the actions of lncRNAs are relatively complex. LncRNA can be a source of miRNA. For example, miR-31 gene is embedded within an intron of the lncRNA LOC554202 and its transcription is regulated by the methylation state of the host gene promoter (Augoff et al., 2012). Morover, lncRNAs can bind to DNA, mRNA and proteins to regulate their expressions or functions (Guttman and Rinn, 2012). The most widely known mechanism is the competitive endogenous RNA (ceRNA), in which way lncRNA acts as a negative regulator of miRNA (Salmena et al., 2011). In recent years, studies have shown that lncRNAs are dynamically expressed in developing and diseased blood vessels, suggesting that lncRNAs have profound biological functions in atherosclerosis (Guo et al., 2019; Simion et al., 2020). LncRNAs can regulate atherosclerosis by influencing the function of vascular cells. For example, lncRNA HOXA11-AS is significantly upregulated in aortic tissue of atherosclerotic mice and oxidized low-density lipoprotein (ox-LDL)-induced endothelial cells. HOXA11-AS knockdown attenuates endothelial injuries by directly regulating the miR-515-5p/ROCK1/eNOS axis (Gao et al., 2022). In addition to endothelial cells, lncRNA also plays a role in atherosclerosis by affecting VSMCs and macrophages. For example, lncRNA TUG1 can promote the proliferation of VSMCs by regulating the miRNA-21/PTEN axis (Li et al., 2018b). LncRNA MAARS interacts with HuR to increase macrophage apoptosis in the blood vessels (Simion et al., 2020). What’s more, lncRNA kcnq1ot1 can compete with miR-452-3p to promote macrophage lipid accumulation and accelerate the development of atherosclerosis (Yu et al., 2020a).

### 2.3 circRNAs and atherosclerosis

CircRNAs are closed circular molecules, which distinguishes them from other linear RNA molecules. CircRNAs were originally considered as by-products of mRNA cleavage, but now they are thought to be stable and functional ncRNAs (Chen, 2016). Compared to miRNAs, circRNAs are less studied ncRNAs in atherosclerosis. Still, there are studies that have shown circRNAs can regulate the fate and function of atherosclerosis-associated cells, including endothelial cells, macrophages, and VSMCs. As with lncRNAs, circRNAs can also compete with miRNAs as ceRNAs, which is the mostly investigated mechanism (Ren et al., 2021). In endothelial cells, a study demonstrated that circ-RELL1 plays a pro-inflammatory role in endothelial cells by directly binding to miR-6873-3p and subsequently activating NF-κB signaling pathway (Huang et al., 2020). Circ_0086296 induces aberrant endothelial cell phenotypes by spongesizing miR-576-3p, resulting in severe atherosclerotic lesions (Zhang et al., 2022). In VSMCs, circRNA-0044073 promotes the proliferation and invasion of VSMCs by targeting miR-107 and activating the JAK/STAT signaling pathway (Shen et al., 2019). In macrophages, overexpression of circ_0004104 results in dysregulation of atherosclerosis-related genes in THP-1-derived macrophages (Wang et al., 2019). It is noticed that the role of circRNAs in atherosclerosis has rarely been studied, which may become a research hotspot in the future.

Since the role of ncRNAs in atherosclerosis is emerging, they have been considered as potential drug targets in developing therapeutic agents. As we know, traditional Chinese medicine has a long history of treating atherosclerosis in China. In particular, studies have shown that the monomers extracted from traditional Chinese medicine are the main functional components that possess anti-atherosclerotic activity, and these activities can be mediated by ncRNAs.

## 3 Active monomers of traditional Chinese medicine relieve atherosclerosis by regulating ncRNAs

Nowadays, the researches about the regulation of atherosclerosis by active monomers of traditional Chinese medicine are tremendous. However, the drug targets of traditional Chinese medicine remain unclear, which affects the clinical application of these medicine. It is clear that ncRNAs appear to be important players during atherosclerosis and important targets of traditional Chinese medicine. Therefore, it is particularly important to discover the mechanism by which the active monomers of traditional Chinese medicine relieve atherosclerosis through ncRNAs.

### 3.1 Geniposide

Geniposide, an iridoid glucoside, is the main active ingredient of *Gardenia jasminoides* J. Ellis. Geniposide exhibits a variety of anti-inflammatory and anti-oxidative functions and has good therapeutic effects on cardiovascular diseases (Fu et al., 2012b). A study has found that geniposide treatment reduces lipid levels and plaque size in the mouse model of atherosclerosis. Mechanistically, geniposide downregulates miR-101 to upregulate mitogen-activated protein kinase phosphatase-1 (MKP-1) and suppresses the production of inflammatory factors in macrophages (Cheng et al., 2019). MiR-21 has been shown to play an important role in regulating inflammatory responses by targeting phosphatase and tensin homolog (PTEN) (Sheedy, 2015; Li et al., 2022). A study established a endothelial cell injury model by using ox-LDL and found geniposide protects endothelial cells from ox-LDL-induced injury by inhibiting oxidative stress and inflammation, and these effects are partly due to the enhancement of the miR-21/PTEN pathway (Zhou et al., 2020). Taken together, miR-101 and miR-21 are involved in the anti-inflammatory effect of geniposide in the setting of atherosclerosis.

### 3.2 Astragaloside IV

Astragaloside IV is a saponin isolated from *Astragalus membranaceus* (Fisch.) Bunge, which has excellent cardioprotective effects (Tan et al., 2020). Astragaloside IV has been reported to protect endothelial cells from oxidative damage caused by ox-LDL through regulating the LOX-1/NLRP3 signaling pathway (Qian et al., 2019a). Recently, a study found that circ_0000231 is the key downstream target of astragaloside IV, which regulates miR-135a-5p to target chloride intracellular channel 4 (CLIC4), and contributes to the protective role of astragaloside IV in ox-LDL-induced endothelial cell injury (Shao et al., 2021). CLIC4 is also a protein associated with endothelial cell apoptosis (Zhang et al., 2020b), indicating astragaloside IV may also inhibit endothelial cell apoptosis by regulating CLIC4 through circ_0000231. Several miRNAs have been shown to be the targets of astragaloside IV. For example, astragaloside IV can protect cardiomyocytes from hypoxia-induced injury by downregulating miR-23a and miR-92a (Gong et al., 2018). ABCA1, a membrane transporter that mediates cholesterol efflux (Chen et al., 2022), has been proved to be a target of miR-33a (Gao et al., 2018). A study has found that astragaloside IV can promote cholesterol efflux in macrophages and inhibit atherosclerosis through regulating miR-33a/ABCA1 pathway (Qin et al., 2018). The serum miR-17-5p is elevated in patients with atherosclerosis and miR-17-5p knockdown can alleviate atherosclerotic lesions and inhibit the proliferation and migration of VSMCs by directly up-regulating very low density lipoprotein receptor (VLDLR), or indirectly regulate VLDLR by affecting proprotein convertase subtilisin kexin 9 (PCSK9) (Tan et al., 2017). Astragaloside IV has been shown to downregulate miR-17-5p and further affect VLDLR expression, thus inhibiting vascular inflammation (Qin et al., 2022). In addition, lncRNA H19 has also been reported to mediate astragaloside IV’s anti-atherosclerotic effect. H19 negatively regulates dual-specificity phosphatase 5 (DUSP5) expression and represses DUSP5/ERK1/2 axis (Tao et al., 2016). Astragaloside IV could attenuate autophagy and mineralization of VSMCs in atherosclerosis by up-regulating H19 and inhibiting DUSP5 (Song et al., 2019). In summary, astragaloside IV can regulate the function of endothelial cells, VSMCs, and macrophages in atherosclerosis by targeting multiple miRNAs, lncRNAs and circRNAs. Therefore, it can be expected that astragaloside IV can exert an excellent anti-atherosclerotic effect through ncRNAs in the clinic.

### 3.3 Notoginsenoside R1

Notoginsenoside R1, the monomer extracted from *Panax notoginseng* (Burkill) F.H.Chen, has a unique effect of promoting blood circulation and has been used on clinical treatment of cardiovascular diseases (Lei et al., 2022). Myeloid differentiation primary response gene 88 (MyD88) is an important immunoregulatory factor, and studies have found that inhibiting MyD88 has a good effect on diabetes (Androulidaki et al., 2018). Notoginsenoside R1 was found to relieve high glucose-induced endothelial cell inflammation and oxidative stress by downregulating the MyD88 via up-regulating miR-147a (Li and Huang, 2021). The Toll-like receptor 4 (TLR4)/Nuclear factor-κB (NF-κB) pathway participates in oxidative stress and induces atherosclerosis in ApoE^−/−^ mice by up-regulating inflammatory cytokines (Tang et al., 2015). A study revealed that notoginsenoside R1 could upregulate the expression of miR-221-3p to target TLR4/NF-κB pathway, thereby inhibiting ox-LDL-induced endothelial cell apoptosis, oxidative stress, and inflammation (Zhu et al., 2020). Notoginsenoside R1 may also play a role in delaying senescence of endothelial cells. Notoginsenoside R1 can decrease the expressions of miR-34a and p53, while increase the expression of SIRT1, thus enhancing the intracellular superoxide dismutase (SOD) activity and cell proliferation capacity in hydrogen peroxide-induced endothelial cell aging model (Lai et al., 2018). These studies suggest that notoginsenoside R1 has a strong and multifaceted endothelial protective effect through regulating ncRNAs.

### 3.4 Tanshinone IIA, salvianolic acid B, tanshinol

Tanshinone, extracted from the traditional Chinese medicine *Salvia miltiorrhiza* Bunge, is a fat-soluble phenanthrene quinone compound with bacteriostatic effect (Wang et al., 2017). Among tanshinone, tanshinone IIA has been clinically proved to have a more significant effect on cardiovascular diseases, especially its anti-inflammatory effect on macrophages. Tanshinone IIA reduces the production of inflammatory factors and adipogenesis in macrophages by up-regulating miR-130b and downregulating WNT5A, thereby relieving the development of atherosclerosis (Yuan et al., 2020). Previous studies have demonstrated that miR-712 is involved in atherosclerosis-related pathological processes, such as VSMCs calcification and endothelial cell inflammation (Son et al., 2013). Tanshinone IIA can inhibit VSMCs inflammation and proliferation by inhibiting miR-712-5p (Qin et al., 2020). KLF4, an evolutionarily conserved zinc-finger-containing transcription factor, is thought to induce M2 and inhibit M1 macrophage polarization (Liao et al., 2011). A study found that the miR-375/KLF4 pathway plays a dominant role in macrophage polarization and autophagy, and tanshinone IIA could activate KLF4 by inhibiting miR-375, leading to enhanced autophagy as well as M2 polarization of macrophages (Chen et al., 2019a). Tropomyosin 1 (TPM1), as a target gene for miR-21-5p (Baker, 2011), is involved in the formation, stabilization and regulation of cytoskeletal actin fibers (Gunning et al., 2015). It was found that tanshinone IIA could downregulate miR-21-5p and then target TPM1, which helps to inhibit the proliferation and migration of VSMCs (Jia et al., 2019).

Salvianolic acid B, a water-soluble compound extracted from *S. miltiorrhiza* Bunge, has been used to treat cardiovascular diseases for hundreds of years. MiR-146a is involved in the regulation of cell proliferation, migration, differentiation, and apoptosis (Cheng et al., 2013). A study has found that salvianolic acid B can inhibit angiotensin II-induced VSMCs proliferation and improve carotid artery ligation-induced neointimal hyperplasia by downregulating miR-146a (Zhao et al., 2019).

Tanshinol is also an active ingredient isolated from *S. miltiorrhiza* Bunge which has the effect of protecting vascular endothelium and reducing atherosclerosis (Song et al., 2014). MiR-26a has been proved to have anti-apoptotic effect on endothelial cells (Zhang et al., 2015). A study found that tanshinol inhibits apoptosis of endothelial cells and reduces atherosclerotic lesions via decreasing lncRNA TUG1 and increasing miR-26a in endothelial cells (Chen et al., 2016).

### 3.5 Genkwanin

Genkwanin is one of the major non-glycosylated flavonoids extracted from *Daphne genkwa* Siebold & Zucc. (Bao et al., 2019). MKP-1 is a key negative regulator of macrophage signaling in response to inflammatory stimulis and is responsible for shutting down the production of pro-inflammatory cytokines (Chen et al., 2002; Chi et al., 2006). Genkwanin was proved to potently decrease the production of proinflammatory mediators through downregulating miR-101 and increasing MKP-1 (Gao et al., 2014).

### 3.6 Dihydromyricetin

Dihydromyricetin, a bioactive flavonoid isolated from *Ampelopsis cantoniensis* var. *grossedentata* Hand. -Mazz. and *Ziziphus jujuba* Mill., has been found to have a wide range of pharmacological activities, such as anti-inflammatory (Sun et al., 2021), analgesic (Guan et al., 2019), anti-tumor (Chen et al., 2020) and hepatoprotective effects (Silva et al., 2020). Nitric oxide (NO), produced by endothelial nitric oxide synthase (eNOS), plays a key role in maintaining endothelial function, and impaired NO biosynthesis is a hallmark of atherosclerosis (Tousoulis et al., 2012; Cyr et al., 2020). There is evidence that overexpression of dimethylarginine dimethylaminohydrolase-1 (DDAH1) increases NO production through an asymmetric dimethylarginine (ADMA) manner (Pope et al., 2009). Studies suggested that dihydromyricetin treatment inhibits atherosclerotic lesion formation by increasing NO production by endothelial cells. MiR-21 expression can be reduced by dihydromyricetin in endothelial cells, which increases DDAH1 and reduces ADMA levels (Yang et al., 2018; Yang et al., 2020). Taken together, dihydromyricetin activates endothelial DDAH1/ADMA/eNOS/NO pathway by reducing miR-21, which relieves the pathogenesis of atherosclerosis.

### 3.7 Sulforaphane

Sulforaphane is an isothiocyanate, which is produced by the conversion of glucoraphanin through the myrosinase (Vanduchova et al., 2019). Sulforaphane, a potent antioxidant, is primarily found in several Brassicaceae vegetables, such as broccoli, cauliflower, cabbage, and Brussels sprouts. Sulforaphane has often been shown to protect cells from oxidative stress in cardiomyocytes and neural cells (Guerrero-Beltran et al., 2012). The nuclear factor erythroid-2-related factor 2 (Nrf2), a basic leucine zipper transcription factor that serves as a defense mechanism against oxidative stress, has been shown to be activated by sulforaphane (Bai et al., 2015; Houghton et al., 2016). SIRT1 is a potential target gene of miR-34a (Yamakuchi et al., 2008) and the role of the miR-34a/SIRT1 axis in oxidative stress-induced cellular damage has been demonstrated (Guo et al., 2017). Sulforaphane was found to protect endothelial cells from oxidative stress by regulating the miR-34a/SIRT1 axis through upregulation of Nrf2 (Li et al., 2021c). In addition, a study found that sulforaphane can reduce lipopolysaccharide-induced cell damage and oxidative stress by inhibiting miR-155 in microglia (Eren et al., 2018). MiR-155 was proved to aggravate the carotid atherosclerotic lesion through induction of endothelial cell apoptosis and activation of inflammasome in macrophages (Yin et al., 2019b). Therefore, it is possible that sulforaphane may limit the formation of atherosclerotic lesions by inhibiting miR-155, but clearly, more studies are needed to confirm this hypothesis.

### 3.8 Cyanidin-3-O-glucoside

Anthocyanins are abundant natural water-soluble pigments, which are relatively rich in the skin of *Glycine max* (L.) Merr. These compounds have been shown to exert antioxidant and anti-inflammatory properties (Zhang et al., 2020a). Cyanidin-3-O-glucoside is one of the most abundant anthocyanins in nature. A study found that cyanidin-3-O-glucoside treatment not only suppresses blood lipids, but also improves endothelial cell function in a rabbit atherosclerotic model. Mechanistically, these effects are due to decreased expression of miR-204-5p, which leads to the increased expression of SIRT1 and enhanced endothelial cell function (Wang et al., 2020c).

### 3.9 Baicalin

Baicalin, one of the flavonoid compounds, is the main active component of traditional Chinese medicine *Scutellaria baicalensis* Georgi (Li et al., 2009). It has been shown that baicalin can alleviate the development of atherosclerosis through its anti-adipogenic, anti-inflammatory and antioxidant effects (Wu et al., 2018). The expression of miR-126 was found to be reduced in the peripheral blood of atherosclerotic patients (Jiang et al., 2014). High mobility group box 1 protein (HMGB1) is an essential facilitator of atherosclerosis by enhancing inflammation (Boteanu et al., 2017). It has been found that baicalin induces the upregulation of miR-126-5p and the downregulation of HMGB1, inhibiting ox-LDL-induced proliferation and migration of VSMCs (Chen et al., 2019b).

### 3.10 Curcumin

Curcumin is the main active ingredient of *Curcuma longa* L. and is mainly extracted from dried powdered turmeric. There is evidence that curcumin can modulate the inflammatory response and alleviate inflammatory diseases like atherosclerosis (Hasan et al., 2014; Chen et al., 2015). Studies found that the activated miR-126-3p from endothelial cells and VSMCs plays a key role in reducing vascular calcification (Zeng et al., 2021) and curcumin upregulates miR-126-3p expression (Li et al., 2021b). Therefore, we infer that miR-126-3p may be one of the targets of curcumin in the treatment of atherosclerosis. LncRNA MIAT has been shown to aggravate the atherosclerotic damage through the activation of PI3K/Akt signaling pathway (Sun et al., 2019). A study found that reduced expression of MIAT contributes to the protective effect of curcumin on atherosclerosis. MIAT regulates miR-124 by interacting with enhancer of zeste homolog 2 (EZH2), thereby relieving inflammation in endothelial cells (Ouyang et al., 2022). In addition, curcumin markedly suppresses miR-125a-5p and upregulates SIRT6 in macrophages, thereby regulating the ABCA1 expression and promoting cholesterol efflux of macrophages (Tan et al., 2021).

### 3.11 Epigallocatechin gallate

Epigallocatechin gallate (EGCG) is the most abundant catechin in green tea. EGCG has been shown to has various pharmacological effects including the anti-atherosclerotic effect, which is primarily achieved by promoting intracellular cholesterol efflux in macrophages (Jiang et al., 2012). Recent studies showed that miR-33a is an upstream regulator of ABCA1 (Wijesekara et al., 2012) and EGCG exerts anti-atherosclerotic effect by reducing miR-33a, thereby upregulating ABCA1 and promoting the efflux of cholesterol in macrophages (Yang et al., 2016).

### 3.12 Ginsenoside Rb2

Ginsenoside Rb2, extracted from *Panax ginseng* C.A. Mey., is a commonly used traditional Chinese medicine with antioxidant (Huang et al., 2014), anti-inflammatory (Huang et al., 2017) and anti-apoptotic activities (Gao et al., 2015). In macrophages, ginsenoside Rb2 has been found to exert anti-inflammatory effects by upregulating the expression of an ω-3 fatty acid receptor GPR120 (Huang et al., 2017). A recent study showed that ginsenoside Rb2 can also inhibit endothelial senescence and inflammation. Mechanistically, ginsenoside Rb2 has a specific binding affinity for miR-216a and further attenuates miR-216a-induced inflammatory processes and aging states through the Smad3/IκBα signaling pathway (Chen et al., 2021b).

### 3.13 Paeonol

Paeonol is one of the main active compounds in Tree Peony Bark, which has been found to have anti-inflammatory, anti-thrombotic and antioxidant properties (Fu et al., 2012a; Bao et al., 2013). Paeonol could increase the expression of miR-223 in macrophage-derived exosomes, and after the uptaking of exosomes by endothelial cells, the STAT3 signaling and the related inflammatory response in endothelial cells can be attenuated (Liu et al., 2018). Another study also found similarly protective results of paeonol on endothelial cells in hyperlipidemia-induced atherosclerosis, which is also attributed to cellular uptake of exosomal miR-223 (Shi et al., 2020). Additionally, paeonol also promotes miR-126 expression to inhibit monocyte adhesion to endothelial cells and block the activation of the PI3K/Akt/NF-κB signaling pathway (Yuan et al., 2016). Moreover, miR-21 and its target PTEN also contribute to the protective effects of paeonol on ox-LDL-induced endothelial injury (Liu et al., 2014). MiR-338-3p was proved to be increased in atherosclerotic lesions, and paeonol treatment could downregulates the expression of miR-338-3p and upregulates the expression of Tet methylcytosine dioxygenase 2 (TET2), thereby relieving ox-LDL-induced endothelial cell damage (Yin et al., 2019a; Yu et al., 2020b). Paeonol can also weaken ox-LDL-induced endothelial autophagy through regualting miR-30a/beclin-1 signaling (Li et al., 2018a). Overall, these studies indicate that paeonol has strong endothelial protective effects, which is associated with regulation of various miRNAs and their targets.

### 3.14 Puerarin

*Pueraria lobata* is the dried roots of legumes *P. lobata* (Willd.) Ohwi and *Pueraria thunbergiana* (Siebold & Zucc.) Benth. It is clinically used in the treatment of cardiovascular and cerebrovascular diseases (Wang et al., 2020a). Puerarin, an active monomer in Pueraria lobata, was reported to inhibit the proliferation and inflammation of VSMCs in atherosclerosis by reducing the expression of miR-29b-3p, thereby increasing the expression of insulin-like growth factor 1 (IGF1) (Li et al., 2023). Therefore, puerarin may have a beneficial effect in the treatment of atherosclerosis by regulating miRNA.

## 4 Conclusion and prospects

**FIGURE 1:**
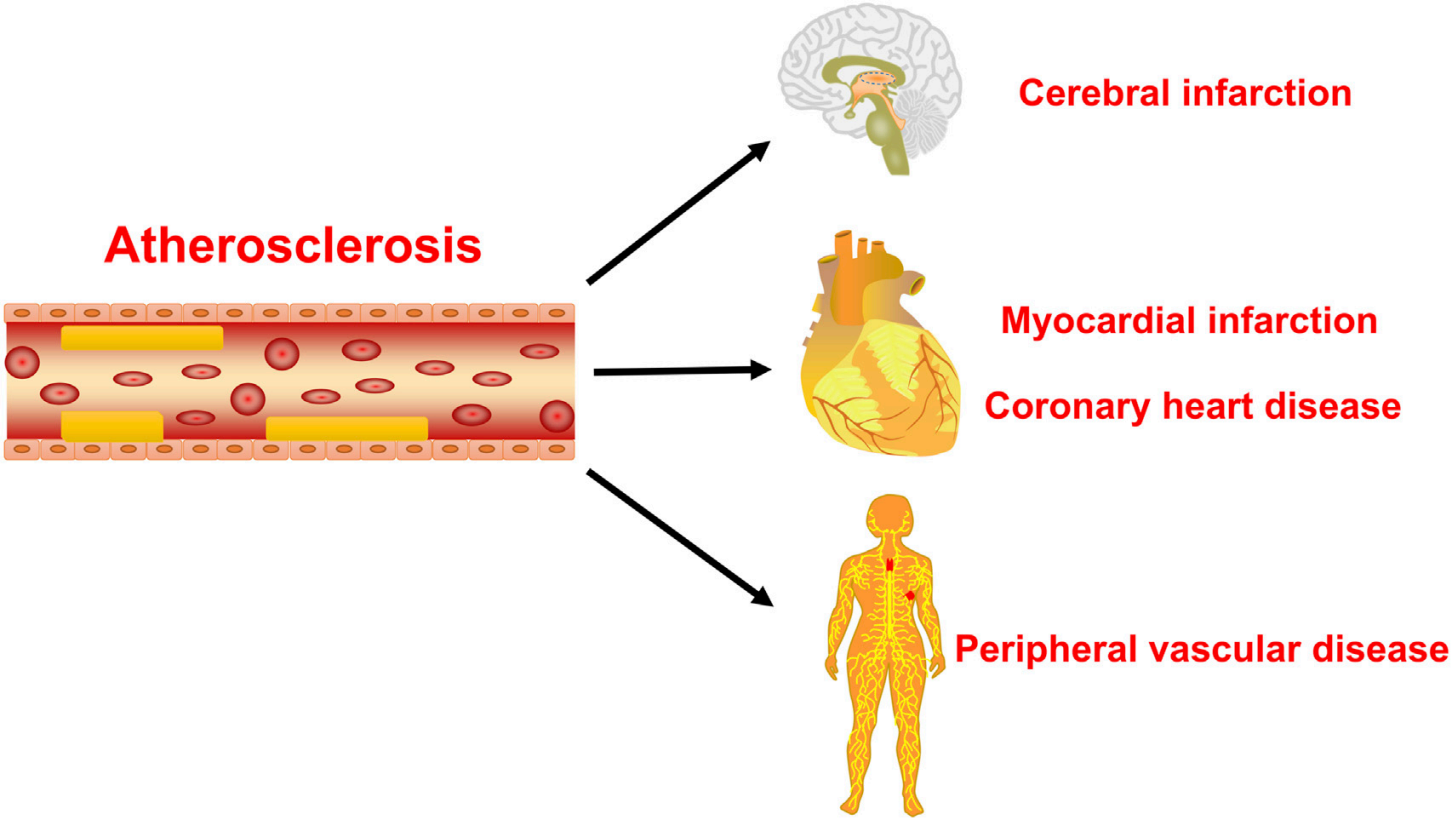
The main hazards of atherosclerosis. The atherosclerosis is the main cause of coronary heart disease, cerebral infarction, and peripheral vascular disease.

**FIGURE 2:**
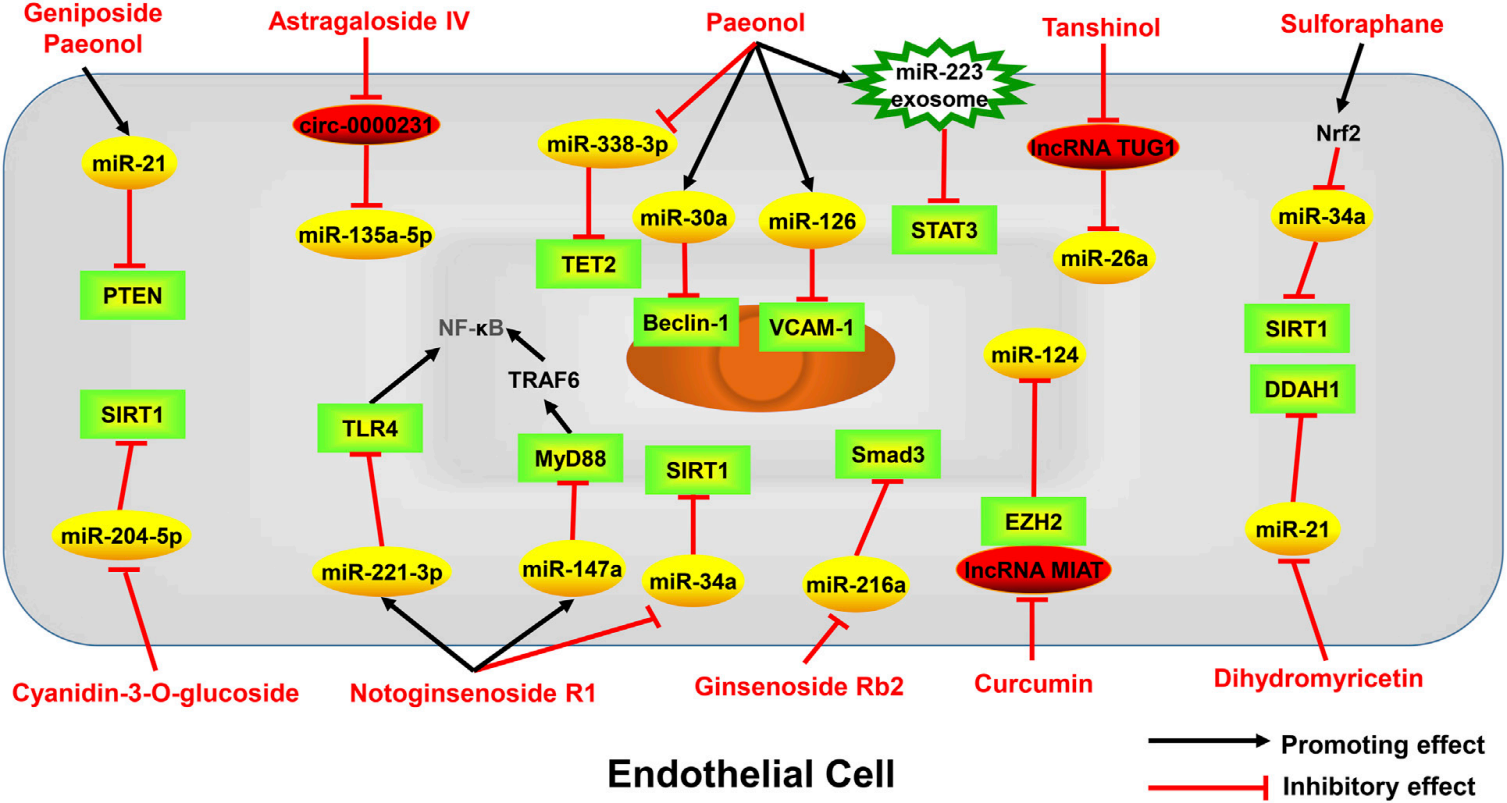
Active monomers of traditional Chinese medicine improve the occurrence and development of atherosclerosis through regulating ncRNAs in endothelial cells. PTEN phosphatase and tensin homolog deleted on chromosome ten, VCAM-1 Vascular cell adhesion molecule-1, TLR4 toll-like receptor 4, MyD88 myeloid differentiation primary response 88, TRAF6 TNF receptor-associated factor 6, NF-κB nuclear factor kappa-B, SIRT1 sirtuin-1, DDAH1 dimethylarginine dimethylaminohydrolase 1, EZH2 enhancer of zeste homolog 2, p53 tumor protein p53, Smad3 sma- and mad-related protein 3, STAT3 signal transducer and activator of transcription 3, TET2 tet methylcytosine dioxygenase 2.

**FIGURE 3:**
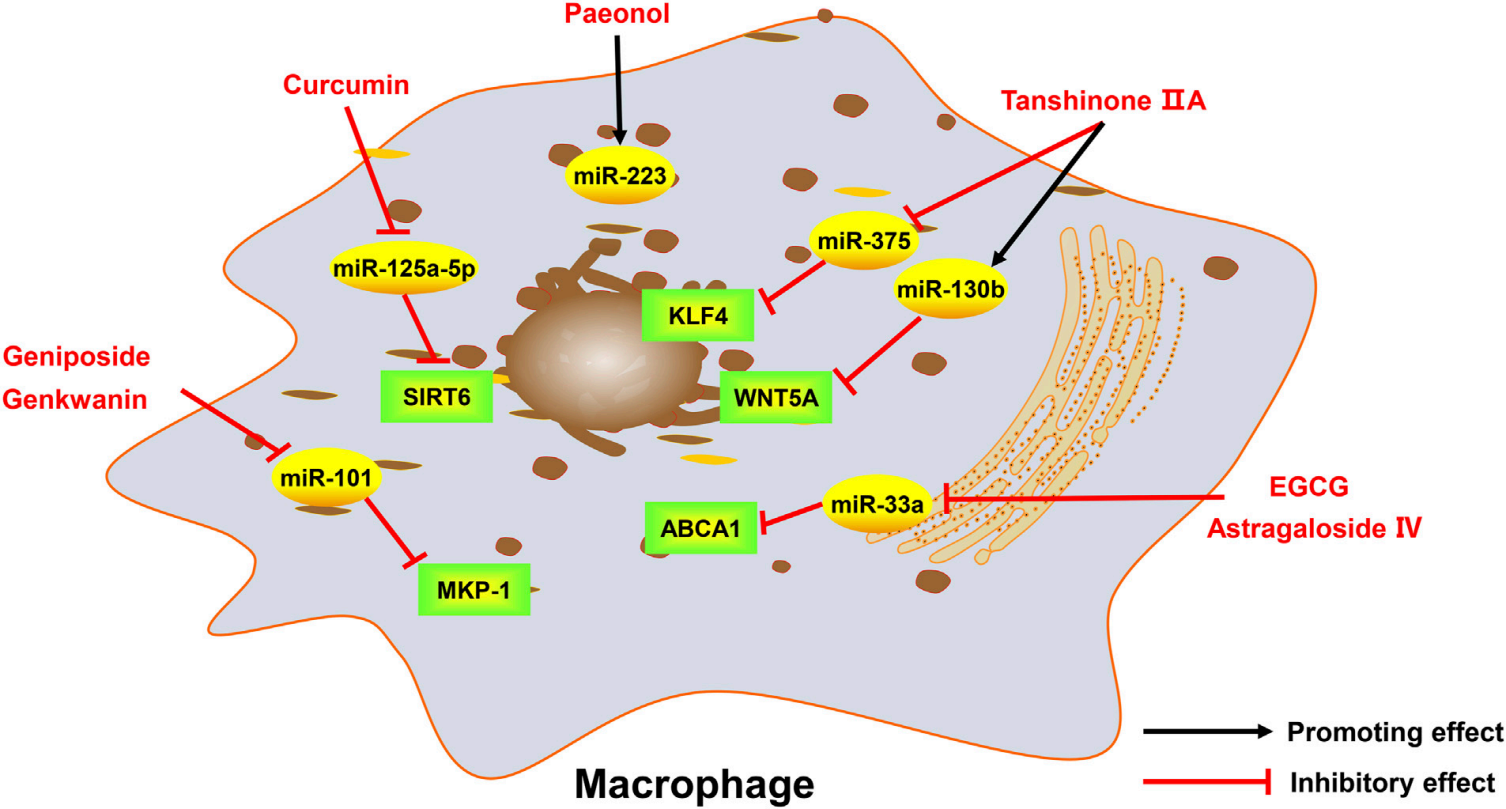
Active monomers of traditional Chinese medicine improve the occurrence and development of atherosclerosis through regulating ncRNAs in macrophages. MKP-1 mitogen-activated protein kinase phosphatase 1, ABCA1 ATP-binding cassette transporter A1, WNT5A wingless/integrated-5A, KLF4 krüppel-like factor 4, SIRT6 sirtuin 6.

**FIGURE 4:**
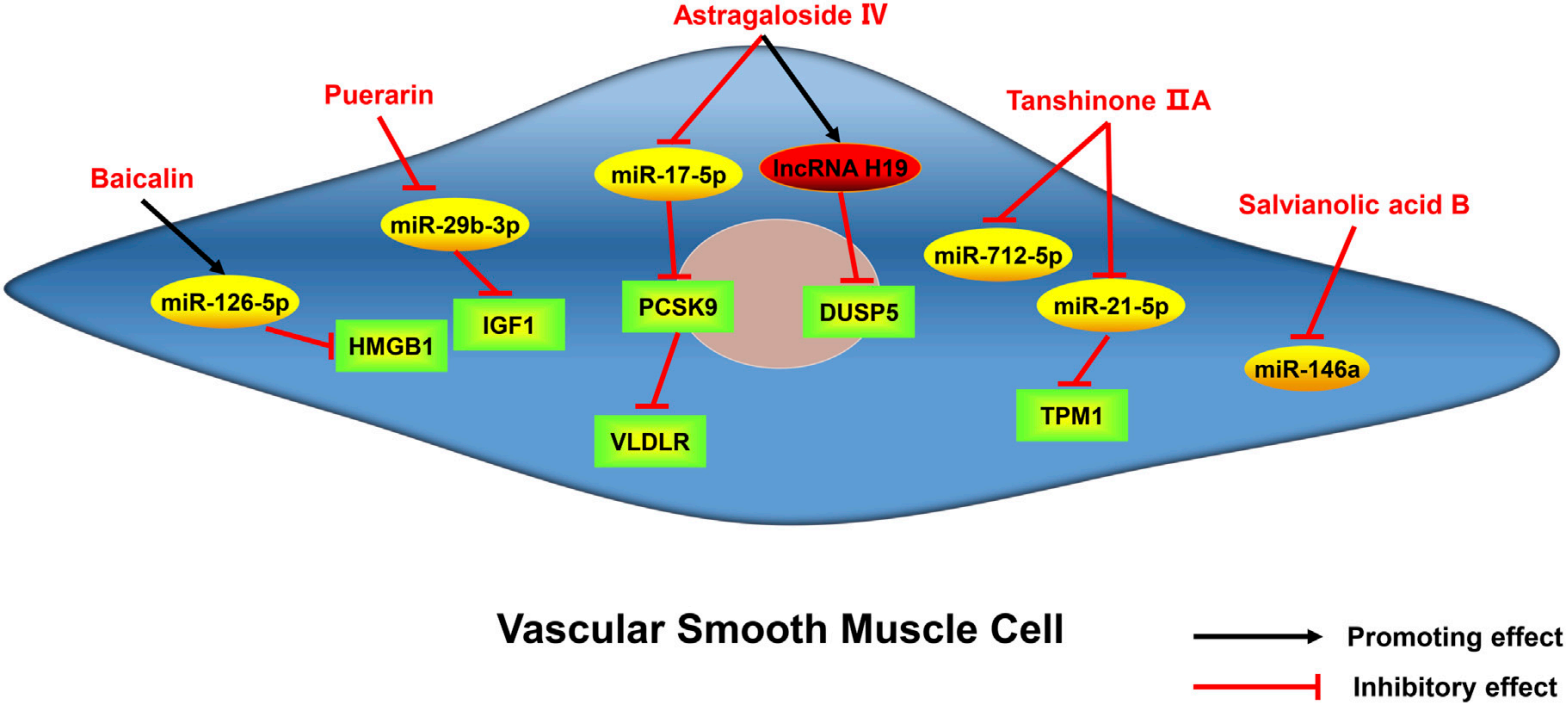
Active monomers of traditional Chinese medicine improve the occurrence and development of atherosclerosis through regulating ncRNAs in vascular smooth muscle cells. DUSP5 dual specificity phosphatase 5, PCSK9 proprotein convertase subtilisin/kexin type 9, TPM1 tropomyosin 1, IGF1 insulin-like growth factor 1, HMGB1 high mobility group box 1 protein, VLDLR very low-density lipoprotein receptor.

**FIGURE 5:**
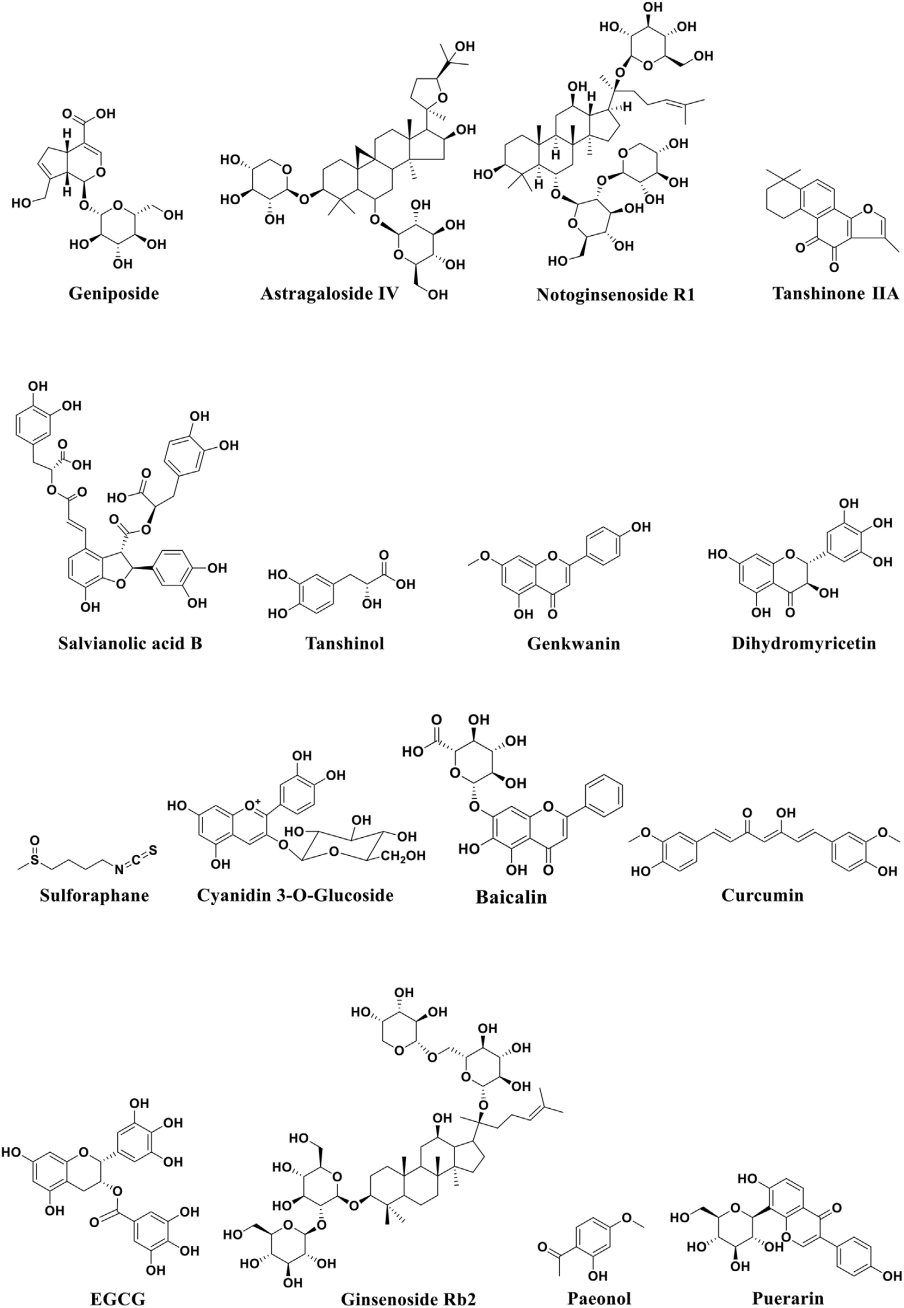
Structural formula of active monomers of traditional Chinese medicine that exhibit anti-atherosclerotic activities.

Atherosclerosis is a major cause of coronary heart disease, cerebral infarction, and some peripheral vascular diseases (Figure 1). With the improvement of living standards, the incidence and mortality of atherosclerosis-induced cardiovascular diseases have increased rapidly in recent years. During the development of atherosclerosis, abnormal expressions of ncRNAs affect the physiological functions of endothelial cells, macrophages, and VSMCs by regulating related signaling pathways or specific proteins. China has a long history of using herbal medicine to treat cardiovascular diseases and the anti-atheroscleroic effects of several herbal medicine are also demonstrated in animal experiments and human studies. The traditional Chinese medicine monomers have recently attracted more attention in the treatment of diseases because they have certain molecular structures, predicted pharmacological effects, less drug-drug interactions, and clear mechanisms of action. Many active monomers derived from traditional Chinese medicines have been evaluated *in vivo* and *in vitro* to ameliorate the development of atherosclerosis by targeting ncRNAs. This article reviews 16 active monomers in traditional Chinese medicine that can improve the development of atherosclerosis by targeting ncRNAs in endothelial cells, macrophages, and VSMCs (Table 1; Figures 2–4). Their structures are shown in Figure 5. Besides monomeric Chinese herbal extracts, Chinese herbal formulas and decoctions have also been proved to treat atherosclerosis by targeting ncRNAs. For example, Tongxinluo Capsule inhibits vascular inflammation and neointimal hyperplasia by inhibiting the expression of miR-155, thereby blocking the feedback loop between miR-155 and TNF-α (Zhang et al., 2014). Alismatis rhizoma decoction, a classic traditional Chinese Medicinal formula used for the treatment of cardiovascular and cerebrovascular diseases, can inhibit the expression of ERK1/2 and miR-17∼92a to inhibit ox-LDL-stimulated VSMCs proliferation (Shen et al., 2020). Among the ncRNAs regulated by active monomers of traditional Chinese medicine, miRNAs are the most studied. However, whether traditional Chinese medicine can exert functions via regulating lncRNAs, circRNAs or other ncRNAs is not well studied and requires more research.

**TABLE 1:** Active monomers of traditional Chinese medicine and their ncRNA targets.

Active monomers	ncRNA	Target genes	Related hallmark	Model	References
Geniposide	miR-101	MKP-1	Inhibits inflammation	*In vivo*: ApoE^−/−^ mice	Cheng et al. (2019)
				*In vitro:* RAW264.7	
	miR-21	PTEN	Inhibits inflammation and oxidative stress	*In vitro*: HUVECs	Zhou et al. (2020)
Astragaloside IV	circ-0000231	miR-135a-5p	Inhibits apoptosis, inflammation, and oxidative stress; Promotes the viability and migration ability	*In vitro*: HUVECs	Shao et al. (2021)
	miR-33a	ABCA1	Promotes the outflow of cholesterol	*In vivo*: ApoE^−/−^ mice	Qin et al. (2018)
				*In vitro*: THP-1	
	miR-17-5p	PCSK9/VLDLR	Inhibits inflammation	*In vivo*: ApoE^−/−^ mice	Qin et al. (2022)
				*In vitro*: VSMCs	
	lncRNA H19	DUSP5	Inhibits autophagy and mineralization	*In vivo*: ApoE^−/−^ mice C57BL/6J mice	Song et al. (2019)
				*In vitro*: HASMCs	
Notoginsenoside R1	miR-147a	MyD88	Inhibits inflammation and oxidative stress	*In vitro*: HUVECs	Li and Huang (2021)
	miR-221-3p	TLR4	Inhibits apoptosis, inflammation, and oxidative stress	*In vitro*: HUVECs	Zhu et al. (2020)
	miR-34a	SIRT1	Delays aging	*In vitro*: HUVECs	Lai et al. (2018)
Tanshinone IIA	miR-130b	WNT5A	Inhibits inflammation and adipogenesis	*In vitro*: THP-1	Yuan et al. (2020)
	miR-712-5p	?	Inhibits inflammation and cell proliferation	*In vitro*: VSMCs	Qin et al. (2020)
	miR-375	KLF4	Enhances autophagy and M2 polarization of macrophages	*In vivo*: ApoE^−/−^ mice	Chen et al. (2019a)
				*In vitro*: RAW264.7	
	miR-21-5p	TPM1	Inhibits proliferation and migration	*In vitro*: HASMCs	Jia et al. (2019)
Salvianolic acid B	miR-146a	?	Inhibits proliferation	*In vivo*: Carotid bifurcation ligated mice	Zhao et al. (2019)
				*In vitro*: VSMCs	
Tanshinol	lncRNA TUG1	miR-26a	Inhibits apoptosis	*In vivo*: ApoE^−/−^ mice	Chen et al. (2016)
				*In vitro*: HAECs, ECV304 cells	
Genkwanin	miR-101	MKP-1	Inhibits inflammation	*In vitro*: RAW264.7	Gao et al. (2014)
Dihydromyricetin	miR-21	DDAH1	Increases NO production and weakens endothelial dysfunction	*In vivo*: ApoE^−/−^ mice	Yang et al. (2018), Yang et al. (2020)
				*In vitro*: HUVECs, THP-1	
Sulforaphane	miR-34a	SIRT1	Reduces oxidative stress	*In vitro*: HUVECs	Li et al. (2021c)
Cyanidin-3-O-glucoside	miR-204-5p	SIRT1	Inhibits inflammation and apoptosis	*In vivo*: Rabbit model of HFD + balloon catheter injury	Wang et al. (2020c)
				*In vitro*: HUVECs	
Baicalin	miR-126-5p	HMGB1	Inhibits proliferation and migration	*ex vivo*:Blood of atherosclerosis patients and healthy people	Chen et al. (2019b)
				*In vitro*: VSMCs	
Curcumin	lncRNA MIAT	EZH2	Inhibits inflammation	*In vivo*: ApoE^−/−^ mice	Ouyang et al. (2022)
				*In vitro*: HUVECs	
	miR-125a-5p	SIRT6	Promotes cholesterol efflux	*In vitro*: THP-1	Tan et al. (2021)
EGCG	miR-33a	ABCA1	Promotes cholesterol efflux	*In vitro*: THP-1	Yang et al. (2016)
Ginsenoside Rb2	miR-216a	Smad3	Inhibits inflammation and aging	*In vitro*: HUVECs, HAECs	Chen et al. (2021b)
Paeonol	miR-223	STAT3	Inhibits inflammation	*In vivo*: ApoE^−/−^ mice	Liu et al. (2018)
				*In vitro*: HUVECs, THP-1	
	miR-223	?	Inhibits inflammation	*In vivo*: SD rats	Shi et al. (2020)
				*In vitro*: RAECs	
	miR-126	VCAM-1	Inhibits monocyte adhesion to endothelial cells	*In vivo*: SD rats	Yuan et al. (2016)
				*In vitro*: VECs isolated from the thoracic aorta of rats	
	miR-21	PTEN	Inhibits inflammation	*In vivo*: SD rats	Liu et al. (2014)
				*In vitro*: VECs isolated from the thoracic aorta of rats	
	miR-30a	Beclin-1	Inhibits autophagy	*In vivo*: SD rats	Li et al. (2018a)
				*In vitro*: VECs isolated from the thoracic aorta of rats	
	miR-338-3p	TET2	Inhibiting apoptosis, inflammation, and oxidative stress	*In vitro*: VECs isolated from the thoracic aorta of mice	Yu et al. (2020b)
Puerarin	miR-29b-3p	IGF1	Inhibits inflammation and proliferation	*In vivo*: ApoE^−/−^ mice	Li et al. (2023)
				*In vitro*: hVSMCs	

MKP-1, mitogen-activated protein kinase phosphatase 1; PTEN, phosphatase and tensin homolog, ABCA1 ATP-binding cassette transporter A1, PCSK9 proprotein convertase subtilisin/kexin type 9, VLDLR, very low-density lipoprotein receptor, KLF4 krüppel-like factor 4, DUSP5 dual specificity phosphatase 5, MyD88 myeloid differentiation primary response 88, TLR4 toll-like receptor 4, SIRT1 sirtuin-1, p53 tumor protein 53, WNT5A wingless/integrated-5A, TPM1 tropomyosin 1, DDAH1 dimethylarginine dimethylaminohydrolase 1, HMGB1 high mobility group box 1 protein, EZH2 enhancer of zeste homolog 2, Smad3 sma- and mad-related protein 3, STAT3 signal transducer and activator of transcription 3,VCAM-1, Vascular cell adhesion molecule-1, IGF1 insulin-like growth factor 1, ApoE^−/−^mice apolipoprotein e-knockout mice, RAW264.7 RAW, 264.7 mouse leukemia macrophage cell line, HUVECs, human umbilical vein endothelial cells; THP-1, human acute monocytic leukemia cell line; VSMCs, vascular smooth muscle cells; HASMCs, human aortic vascular smooth muscle cells; HAEC, human aortic endothelial cells; SD, rats sprague-dawley rats; RAECs, rat aortic endothelial cells; VECs, vascular endothelial cells; hVSMCs, human vascular smooth muscle cells, TET2 tet methylcytosine dioxygenase 2.

NcRNAs are the most abundant transcripts in cells. In addition to seaching ncRNAs from published papers, we can identify or screen new ncRNAs in the following ways: firsty, we can utilize publicly available genomic and transcriptomic databases, such as Ensembl, NCBI, and UCSC to identify regions of the genome that are transcribed but not coding for proteins, indicating potential ncRNAs; additionally, high-throughput sequencing like RNA-Seq can be used to identify novel transcripts, including potential ncRNAs; furthermore, we can also compare the genomic sequences across various species in order to find conserved non-coding regions, which may potentially serve as ncRNAs; besides, machine learning algorithms based on sequence features and structural properties of ncRNAs can also be used to predict potential novel ncRNAs. After discovering new ncRNAs, techniques like CRISPR/Cas9, RNA interference, qRT-PCR, Northern blotting, *in situ* hybridization and other functional assays can be used to identify the specific biological functions of the ncRNAs. It can be expected that future studies will find more and more ncRNAs that related to atherosclerosis and these ncRNAs can be used as drug targets for development of anti-atherosclerotic drugs.

Over the past decades, substantial effort has been made towards the clinical application of RNA-based therapeutics, such as small interfering RNAs and antisense oligonucleotides. However, since the hurdle of immunogenicity, specificity, and delivery, some studies demonstrated limited efficacy or toxicity of ncRNAs-based therapies. Therefore, traditional Chinese medicine may become alternative drugs by targeting ncRNAs to treat atherosclerosis. It is worth noting that most studies suggest that traditional Chinese medicine treats atherosclerosis by targeting a specific ncRNA. However, the mechanism of ceRNA suggests that ncRNAs may have complex interactions in cells. What’s more, a ncRNA may also have multiple targets. Therefore, we should further explore the anti-atherosclerotic mechanisms and clinical safety of these traditional Chinese medicine in more detail. It is hoped that by studying the regulation of ncRNAs by traditional Chinese medicine, it will provide theoretical support for the future research and clinical application of traditional Chinese medicine for treatment of atherosclerosis.

While many traditional Chinese medicines have therapeutic effects on atherosclerosis, some research has also identified potential side effects of certain Chinese herbs that can exacerbate atherosclerosis. For example, a moderate dosage of marijuana proves highly efficient in alleviating chronic pain (Carter et al., 2015), but marijuana can also cause cardiovascular side effects, such as endothelial dysfunction and atherosclerosis (Feng et al., 2022). Proanthocyanidin A1 can promotes the production of platelets to ameliorate chemotherapy-induced thrombocytopenia (Wang et al., 2022) and TMEA, a polyphenol in *Sanguisorba officinalis* L., can facilitate megakaryocyte differentiation and platelet production (Li et al., 2021a). However, the increased platelets can raise the risk of blood clot formation in patients with atherosclerosis (Barrett et al., 2019). Therefore, when patients have concurrent atherosclerosis, the use of these Chinese herbal medicines should be avoided. Furthermore, studying the ncRNAs that may mediate these effects is of significant importance, but this field is still lacking in research and requires further investigation.

## Figures and Tables

**Table:** 

**ABCA1**	ATP-binding cassette transporter A1
**ADMA**	Asymmetric dimethylarginine
**ApoE** ^**−/−**^ **mice**	Apolipoprotein e-knockout mice
**ceRNA**	Competitive endogenous RNA
**circRNAs**	Circular RNAs
**CLIC4**	Chloride intracellular channel 4
**DDAH1**	Dimethylarginine dimethylaminohydrolase-1
**DUSP5**	Dual-specificity phosphatase 5
**EGCG**	Epigallocatechin gallate
**eNOS**	Endothelial nitric oxide synthase
**EZH2**	Enhancer of zeste homolog 2
**HAEC**	Human aortic endothelial cells
**HASMCs**	Human airway smooth muscle cells
**HA-VSMCs**	Human aortic vascular smooth muscle cells
**HMGA2**	High mobility group AT-hook 2
**HMGB1**	High mobility group box 1 protein
**HMG-CoA**	Hydroxymethylglutaryl-CoA
**HUVECs**	Human umbilical vein endothelial cells
**hVSMCs**	Human vascular smooth muscle cells
**IGF1**	Insulin-like growth factor 1
**KLF4**	Krüppel-like factor 4
**lncRNAs**	Long non-coding RNAs
**MCP-1**	Monocyte chemoattractant protein 1
**miRNAs**	MicroRNAs
**MKP-1**	Mitogen-activated protein kinase phosphatase-1
**MyD88**	Myeloid differentiation primary response gene 88
**ncRNAs**	Non-coding RNAs
**NF-κB**	Nuclear factor-κB
**NO**	Nitric oxide
**Nrf2**	Nuclear factor erythroid-2-related factor 2
**ox-LDL**	Oxidized low-density lipoprotein
**p53**	Tumor protein 53
**PCSK9**	Proprotein convertase subtilisin kexin 9
**PTEN**	Phosphatase and tensin homolog
**RAECs**	Rat aortic endothelial cells
**RAW264.7**	RAW 264.7 mouse leukemia macrophage cell line
**SD rats**	Sprague-dawley rats
**SIRT1**	Sirtuin 1
**SIRT6**	Sirtuin 6
**Smad3**	Sma- and mad-related protein 3
**SOD**	Superoxide dismutase
**STAT3**	Signal transducer and activator of transcription 3
**TET2**	Tet methylcytosine dioxygenase 2
**THP-1**	Human acute monocytic leukemia cell line
**TLR4**	Toll-like receptor 4
**TNF-α**	Tumor necrosis factor-α
**TPM1**	Tropomyosin 1
**TRAF6**	TNF receptor-associated factor 6
**VCAM-1**	Vascular cell adhesion molecule-1
**VECs**	Vascular endothelial cells
**VLDLR**	Very low-density lipoprotein receptor
**VSMCs**	Vascular smooth muscle cells
